# Adverse Childhood Experiences, Domestic Violence and Substance Misuse: An Action Research Study on Routine Enquiry and Practice Responses

**DOI:** 10.3389/fpsyt.2022.892849

**Published:** 2022-07-12

**Authors:** Sarah Morton, Megan Curran, Mary Barry O'Gorman

**Affiliations:** ^1^School of Social Policy, Social Work and Social Justice, University College Dublin, Dublin, Ireland; ^2^Center on Poverty and Social Policy, School of Social Work, Columbia University, New York, NY, United States; ^3^Cuan Saor Women's Refuge, Clonmel, Ireland

**Keywords:** substance use, domestic violence, adverse childhood experience (ACE), trauma informed responses, organizational change, practitioner change

## Abstract

The long-term impacts of Adverse Childhood Experiences (ACEs) are of increasing interest to researchers and practitioners, including the effectiveness of screening for ACEs to improve health and social outcomes. Despite a focus on implementing such practices, there has been little focus on ACEs experiences for women experiencing domestic violence and substance use, or consideration of practice responses around ACEs routine enquiry for domestic violence and related services. The Irish study discussed in this paper used an action research approach to implement ACEs routine enquiry within a domestic violence service for women accessing the service (*n* = 60), while also utilizing co-operative inquiry groups for practitioners both within the organization (*n* = 10) and with those working in associated fields of infant mental health, child protection, substance misuse and welfare and community support (*n* = 7). Of the 60 women who completed the ACEs routine enquiry in the study, over one-half (58 per cent) reported experiencing at least two ACEs in their childhood, including one-third of all respondents reporting experiencing four or more; service users reported significant levels of overlap between direct child maltreatment and adverse home environments. Reported parental substance misuse with the home environment was substantially higher than in general population studies. These findings offered early indications of both ACEs prevalence as well the types of ACEs that most define the experiences of the women presenting to a domestic violence service that supports women with substance misuse and other related issues. This paper discusses the ways in which the co-operative inquiry groups used this information and other processes to enhance practitioner, organizational, and inter-agency understanding and service responses. The practitioners felt that this form of ACEs routine enquiry, while not an end in itself, was a useful tool to engage women in conversations about trauma and intergenerational patterns and a basis for developing trauma-informed interventions. We conclude with discussion about: considerations of the risks of “individualizing” women's traumatic experiences; skills and supports for practitioners; and resource implications.

## Introduction

The long-term impacts of Adverse Childhood Experiences (ACEs) are of increasing interest to researchers and practitioners, and consideration is also being given to the effectiveness of routine enquiry for ACEs to improve health and social outcomes. Some attention is also being paid to the links between ACEs and domestic violence, poverty, and substance use in order to inform appropriate health and social care responses (1). As yet we have little data on the ACEs experiences for women experiencing domestic violence and substance use, or consideration of practice responses around ACEs routine enquiry for domestic violence and related services (2). This article presents the findings of an Irish study that used an action research approach to implement ACEs routine enquiry within a domestic violence service that already provides supports to women with active substance misuse issues.

The study of ACEs examines the impact of childhood experiences–particularly stressful ones–on an individual's subsequent life course. The term originated in a US study of the same name conducted by the American health care provider, Kaiser Permanente, and the Division of Violence Prevention in the US Centers for Disease Control and Prevention (CDC) in the mid-1990s. Results, published by Felitti et al. (3), found a strong interrelationship between adverse childhood experiences and severe chronic disease and premature death in adulthood, effectively launching what is now a growing body of research and evidence-based practice. The wide range of ACEs literature now interrogates the effects of physical and emotional treatment, familial relationships, and home environments experienced as a child on individuals' future emotional, health, education, financial outcomes, as well as the potential for intergenerational transmission or replication of similar trauma on their own children. It is important to note that ACEs theory and practice sit alongside or within the context of other approaches and concepts of trauma and intergenerational transmission of trauma, including sociocultural, psychodynamic, biological and family system models (4). For instance, consideration of ACEs has been combined with the concepts of “ghosts” (adversity) and “angels” (benevolence) in the nursery to predict risk and resilience factors across generations (5). It has been argued that uncovering and exploring angels in the childhood experiences of parents may be as vital as identifying adversity (6). Ultimately though, how we intervene and provide adults with greater understanding of their trauma experiences is key (4).

Although ideas about ACEs are at times contested–with some calls for more conceptual clarity and improved measurement (7) and cautions against potential misuse of results (8)–ACEs routine enquiry has produced important results. The current ACEs categories of focus include:

- Child Maltreatment: sexual abuse, physical abuse, verbal abuse.
- Children's Environment: domestic violence, parental separation, mental illness, alcohol abuse, drug abuse, incarceration (9).

Experiencing four or more of these issues is seen to significantly increase the likelihood of a person engaging in future risky behavior, which may lead to a range of poor health outcomes in adulthood (3, 9). ACEs also impact on wider society; for example there may be intergenerational effects and pressures on health and social care agencies, particularly in terms of complex social problems such as substance use and domestic violence (9–11). To date, policy responses tend to focus on prevention and early intervention (12, 13).

The organization in this study, like many domestic violence services, has seen an increase in complex cases, including more overt presentations of substance use issues among women accessing refuge and other support services. ACEs have strong links to both. Research has long identified domestic violence as an issue with the potential for intergenerational patterns (14, 15) violence witnessed or experienced as a child can significantly increase the risk of exposure or perpetration of violence in the home later on (16). Leza et al. (17) review the existing literature on ACEs and substance misuse; they acknowledge that not enough is yet known about protective factors that could mediate this relationship, but that the weight of the available evidence reveals positive associations between ACEs and later substance use disorder (SUD) diagnoses or higher prevalence of ACEs among individuals in substance misuse treatment compared to general populations. Fuller-Thomson et al. (18), for example, find that three particular types of violence-related ACEs–sexual abuse, physical abuse, and exposure to parental domestic violence–have independent relationships to lifetime drug and alcohol dependency.

Brown et al. (19) explore the co-occurrence of substance use and domestic violence, finding that substance use can often be a mediating factor between the violence and earlier ACEs trauma. Experiencing childhood abuse can increase the likelihood of experiencing intimate partner violence as an adult (11) and substance use can often be used to cope with the repeated trauma (20). This can work in the opposite direction as well; childhood abuse can lead to substance use, which can, in turn, increase the risk for domestic violence (for both potential victims and potential aggressors). Violence and substance use become intertwined, but it can be ACEs that drive them both.

There is a gender difference in how this confluence of childhood trauma and adult domestic violence and substance use plays out, with a resulting impact on the services operating at the intersection of these areas. Women are significantly more likely to experience domestic violence as both children and as adults than are men (9). It is important to note, of course, that though research reveals links between ACEs and experiences of being subjected to domestic violence later in life, this does not suggest that the violence is a consequence of women's choices (whether they have experiences of ACEs or not). Women are also more likely to use substances as a means of coping with this violence. Gutierres and Van Puyumbroeck [(10), p. 502] report that 90 per cent in substance misuse treatment have a history of traumatic violence; there is also evidence of a ‘lifespan victimization among women who misuse substances', as the combination of ACEs and substance use puts these women at further risk for future domestic violence and sexual abuse. This has been reiterated by Boppre and Boyer (21) who found that women involved in the criminal justice system often linked their substance use trajectories to experiences of adversity in childhood.

This “lifespan” aspect is key. An important element of ACEs research emphasizes the fact that different types of adverse experiences often co-occur for children (e.g., physical abuse and mental illness in the household and parental separation or child maltreatment alongside intimate partner violence) and the cumulative effect of these interrelationships is critical to understanding ACEs' long-term effects (16, 22). Infant mental health (IMH) is a concept used to describe the social and emotional development of a child from age zero to three (23). It is argued that a poor or stressful relationship with their caregivers will induce stress in the child, potentially affecting their cognitive, physical, emotional and social development over the longer-term (24). Children may be exposed to ACEs in the home, either through maltreatment or through factors in their home environment. Research indicates that parents who misuse substances, for example, may be less able to provide high-quality parenting–a particular risk for forming insecure attachment when children are very young (25).

Infants can also be affected, perinatally, by maternal ACEs (e.g., the adverse childhood experiences of their mother). This has particular salience in Ireland, as Cheong et al. (26) found that ACEs are common among older Irish adults, potentially due to multiple forms of child abuse endemic in state and church run organizations and educational settings (27). McDonnell and Valentino (28) found an intergenerational effect of maternal childhood trauma on infants, in the form of lower birth weight and reducing infant functioning, and a link between higher ACEs scores and maternal depression (pre- and post-partum), with implications for infant attachment. They also found an association between ACEs and pregnancy at an early age, as well as other risky behaviors and links to other forms of social disadvantage. Given these connections, ACEs may offer a useful framework for interventions and services, especially those that deal with complex cases (such as women with children who present with domestic violence experiences and substance use in tandem), because the cases may contain “a complex set of highly interrelated experiences” [(22), p. 773].

A common practice response is to apply a trauma-informed approach to care (29) which has been described “the simple and direct approach of listening and validating [an individual's] experience that shifts from asking,” “What is wrong with you?” to “What has happened to you?” [(30), p. 49, (31), p. 2]. Trust-based relationships and community-based supports can be key components of trauma-informed responses where there are childhood experiences of trauma (32). Leza et al. (17) conclude their scoping review of the connections between ACEs and substance use disorder with specific support for the application of trauma-informed care. Such approaches seek to explore the most appropriate interventions for the individuals and to mitigate any intergenerational effects (33). Najavits (34) work on intervention approaches focuses on building coping skills, acknowledging the inter-relationship between trauma and substance use in women's lives. This concurs with Bath's (32) three components of trauma-informed intervention; safety; trust; and development of coping skills. To identify childhood trauma experiences, some organizations target only those individuals who present to a specific part of the service (for example: in cases of domestic violence cases, those seeking refuge), while others adopt a universal approach, regardless of specialist or intensive need. The timing of routine enquiry can vary–sometimes it is at the point of first contact, others only after establishing a relationship with the service user (33). Within domestic violence organizations, McGee et al. (33) found that crisis mitigation often takes precedence, with the result that ACEs screenings are contingent on the skills of the practitioner at that moment.

Despite the volume of the literature on this topic, ACEs routine enquiry remains challenging and contested. A review of pilot ACEs routine enquiry programmes across a range of sectors in the UK found limitations in delivery caused by lack of organizational expertise, capacity, and commitment (35). This is important in the context of ACEs research where there is criticism of initiatives to introduce ACEs routine enquiry into trauma-informed care in ways that fail to distinguish between potential individual-level impacts (which create potential stigma) instead of group- or population-level application where changes at the level of structure are targeted (8). Training and skill development for staff in responding to historical and ongoing trauma experiences has also been found to be key (36), as well as greater insight in women's recovery pathways where multiple issues exist (37). Given this context, this study sought to (a) identify the level of ACEs for women accessing a domestic violence service; (b) consider and explore trauma-informed responses to women's childhood experiences and the inter-generational transmission of trauma; and (c) consider the role of ACEs routine enquiry and intervention in relation to a range of agencies the domestic violence service work with including those focused on infant mental health (IMH), a key area of work for childcare workers within domestic violence settings.

## Materials and Methods

The study upon which this article is based took place in an organization delivering services to women and children who experience domestic violence and related issues, including substance misuse. Established 25 years ago in a large town in Ireland, the organization provides emergency accommodation, keyworker support, counseling, helpline support, children's interventions and court accompaniment. Over the past decade, the organization has developed specific supports and responses to women who are experiencing problematic substance use, and currently; accommodate women within refuge with substance misuse issues; routinely enquire about substance use issues; support women to access substance use stabilization, treatment, and recovery services; provide harm reduction interventions; and provide substance misuse in-reach for residents (38). In addition, substance misuse responses and interventions are integrated into other services such as the Pattern Change groupwork programme, art therapy and advocacy supports (2).

### Study Design

To meet these aims, an action research approach, involving three phases as seen in Table 1, was taken in a study completed over a nine-month period. The first phase involved the implementation of ACEs routine enquiry for women accessing all aspects of the organization's services (*n* = 60 service user participants) using the ten-question ACEs questionnaire from the US Centers for Disease Control and Prevention short ACEs tool (39). The second phase, undertaken concurrently, was a series of co-operative inquiry groups facilitated with domestic violence service staff and designed to support their implementation of the ACEs routine enquiry with service users and their development of responses to women who completed the routine enquiry. The third phase involved the facilitation of an inter-agency co-operative inquiry group with external community service partners on the potential to integrate ACEs into wider inter-agency work, especially where there is a focus on IMH. The study was granted ethical approval by the first author's university.

**Table 1:** Study design.

**Phase 1**	**Phase 2**	**Phase 3**
(Service user implementation)	(Practitioner groupwork support, assessment, and reflection)	
ACEs routine enquiry with women accessing domestic violence organization using ten point questionnaire (39) *n* = 60	Co-operative inquiry group for domestic violence service practitioners. *n* = 10	Co-operative inquiry group for external community partners involved in IMH. *n* = 7

The quantitative element of the study involved the implementation of a ten-question ACEs questionnaire for women accessing the organization over a 4-month period. The questions mirror established ACEs questions from the US Centers for Disease Control and Prevention short ACEs tool and have been used in similar ACEs routine enquiry implementation to measure childhood exposure to forms of abuse and household dysfunction (39). All questions were yes/no; four questions focused on direct maltreatment experienced as a child (e.g., abuse experienced themselves) and six focused on their home environment as a child (e.g., abuse experienced by other members of the household or substance misuse by household members, among other things). For example, direct maltreatment questions ranged from emotional (did a parent or other adult…act in a way that made you feel worthless or scared) to physical (did a parent or other adult push, grab, slap…or ever hit you so hard you…were injured?/touch you or make you touch their body in a sexual way…?) to neglect [did your parent(s) make you go without enough food or drink, clean clothes, or a clean and warm place to live for long periods of time?]. Home environment questions asked whether the individual's home as a child saw adults with mental health issues, substance misuse, incarceration, physical or emotional abuse, or relationship breakdown. Inclusion criteria were set in regard to women accessing any of the organization's support and refuge services. To be invited to complete the ACEs routine enquiry, women had to: be fully aware of the range of supports offered by service; not be in crisis, which is understood in this practice setting to mean the woman is not dealing with an immediate risk to her safety and wellbeing or that of her children; have attended the service on at least three occasions; and not be significantly affected by current drug or alcohol use. Posters outlining the study and what ACEs routine enquiry consisted of were placed in all public spaces in the organization's building. All women who met the above criteria were invited to participate over a 4-month period. Women also self-selected, asking to participate when they saw the poster. The ACEs routine enquiry was explained by the practitioner. All participants signed forms of consent and were informed that they could change their mind at any time. Participants were informed their ACEs routine enquiry form would be anonymised. Over the 4-month period, sixty completed the ACEs routine enquiry (*n* = 60 women). All of the women invited to complete the ACEs routine enquiry agreed, though one requested to complete it on a different occasion.

### Inquiry Group Procedure

Co-operative inquiry groups are a core action research method that can assist in identifying the needs of those served by the organization and offer the opportunity to explore and respond to presenting problems within practice and organizational contexts (40). Where the subject matter is sensitive, it is important to be attentive to practitioner stressors; for group members to stay emotionally present (41); deal with transference and countertransference (42); and potentially engage in various emotional labor strategies (43) to ensure positive outcomes. The complexity of these processes adds an additional layer to the facilitator's role and group dynamics as a whole (44). For both sets of inquiry groups–the domestic violence service practitioners and the IMH inter-agency practitioners–all group members were involved in the inquiry group structure and design; invited to reflect on their practice in regard to ACEs routine enquiry; and invited to consider and undertake actions in regard to implementation between inquiry group meetings. The practitioners from the domestic violence service had extensive experience, accreditation and professional recognition in areas of domestic and sexual violence, substance use and childhood legacies of trauma, while those in the IMH inter-agency group had a range of professional expertise including social work, infant mental health and substance use. Prior to the fieldwork, a 1-day ACEs routine enquiry training was delivered by an independent training consultant to both the domestic violence organization staff and all members of the IMH group.

Ten of the fourteen practice staff who were invited agreed to participate (*n* = 10 domestic violence practitioners). Three domestic violence service practitioner inquiry groups were run at 4 to 6-week intervals during the ACEs routine enquiry process, with each inquiry group running for approximately 90 min. Each inquiry group was audio recorded, with the consent of participants. Themes for each inquiry group were agreed with participants, and the practitioners were encouraged to describe their practice and skills, as well as explore the experience of enacting ACEs routine enquiry with service users. The members of the inter-agency IMH practitioner inquiry group were drawn from a regional IMH working group. The seven who decided to participate worked in social work, family support, community and substance misuse services (*n* = 7 inter-agency IMH practitioners). Two IMH inter-agency inquiry group sessions were run, with a 4-week interval, with each group running for approximately 90 min. Each inquiry group was audio-recorded with the consent of participants. As the practitioners came from a range of agencies, the discussion and themes for this inquiry group focused on the feasibility and possibility of integrating ACEs routine enquiry into their existing work and organizations. The inquiry groups were facilitated by the lead author, with one IMH inter-agency group co-facilitated by both the first and second authors.

### Data Analysis

The quantitative analysis is based on data from the aforementioned ten-question ACEs surveys completed by sixty women. The questionnaires were administered by the organization's staff, but no personal information was included on survey papers and the data was anonymous to the research team. It was agreed through collaborative discussion with the organization's practitioners that the questionnaire would be explained to women and given to them to complete on their own, with the practitioner staying present only to answer any queries or talk through any of the questions if asked. Where there were literacy or language issues, the practitioner helped the woman to complete the questionnaire by reading the questions or particular questions upon request.

In the analysis, each anonymous respondent was accorded a sum of the number of ACEs experienced. In keeping with the existing empirical literature using ACEs survey data (9, 39), these ACEs totals were also grouped into four ACEs “count” categories: 0 ACEs; 1 ACE; 2-3 ACEs; or 4+ ACEs experienced. The ACEs survey question results were divided into bivariate data (“child maltreatment” vs. “childhood home environment”) and descriptive analysis on the prevalence of each specific type of adverse experience within those categories was conducted. Results from the study sample were also compared to the results of ACEs studies in Wales (9) and the United States (3, 45) in order to understand the ways in which the prevalence (and types) of ACEs experienced by women accessing domestic violence services differed from the ACEs experience of broader populations in primary care settings. The order of prevalence of individual ACEs types self-reported among the survey participants was also specifically noted, along with the associative patterns among the most common ACEs type to other ACEs experienced, in order to identify trends within this particular cohort of women and to inform local practitioners moving forward.

The practitioner inquiry groups generated a good deal of qualitative data which was analyzed thematically (46) to explore key issues emerging from the data. To reduce the data and make it more manageable (47), two levels of coding, open and axial (48) were conducted. The first step allowed for categories to be identified and assigned to elements of the recorded material and the second step allowed for relationships between the categories to be established (48). The practitioners did discuss their engagement with clients and reported on anonymized interactions within the inquiry groups. It had been agreed that in these discussions, all identifying client details would also be absent from the discussion. The practitioners were anonymized within the analysis stage.

## Results

### ACEs Routine Enquiry: Questionnaire Findings

The survey results reveal ACEs to have a significant presence among the domestic violence organization's service users. The mean ACEs score for the women surveyed was 2.7. While 18 per cent of service users reported having experienced no ACEs in their childhood, over one-half (58 per cent) of the 60 service users who participated experienced at least two ACEs in their childhood. One-third of all respondents reported experiencing four or more ACEs. Service users also reported significant levels of overlap between direct child maltreatment and adverse home environments. Reported parental substance misuse with the home environment was substantially higher than in general populations studies (1).

Table 2 identifies the prevalence of each type of ACEs across the service users participating in the routine enquiry process. Half of the service users surveyed experienced verbal and/or emotional abuse as a child. Over half (53 per cent) lived in a household where substances were misused (40 per cent with alcohol abuse and 13 per cent with drug misuse). Mental illness^1^ in the household was the third most common type of ACE experienced by service users. Violence in the household, in the form of physical abuse of the child or physical abuse of other family members, affected one-third of respondents. Sexual abuse and parental breakup follow closely thereafter, affecting at least one-quarter of service users surveyed.

**Table 2:** Prevalence of ACEs, by type, experienced by study service users (%) (*n* = 60) (2019).

**Child emotional abuse**	**50%**
**Alcoholism (in household)**	**40%**
**Mental illness (in household)**	**38%**
**Child physical abuse**	**32%**
**Violence (in household)**	**32%**
**Child sexual abuse**	**27%**
**Parental breakup**	**25%**
**Drug misuse (in household)**	**13%**
**Incarceration (in household)**	**8%**
**Child neglect**	**7%**

*Respondents able to select as many as applied*.

Furthermore, the most prevalent ACEs experienced did not happen in isolation – each strongly overlapped with one another. Of all the respondents who reported experiencing emotional/verbal abuse as a child (*n* = 30), 60 per cent also reported alcoholism in their household, 53 per cent reported having experienced physical abuse, 50 per cent reported domestic violence in the household, 46 per cent reported mental illness in the household, and 40 per cent reported experiencing sexual abuse. ACEs routine enquiry is best able to capture exposure and patterns of association; it offers much less in terms of identifying the intensity or duration of adverse experiences and does not offer a causal link between ACEs in childhood and later life outcomes. In terms of quantitative data, the sample size (*n* = 60) is also limited and representative of one organization. It provides valuable descriptive information on the cohort of service users who participated, but is not necessarily a portrait of the whole population of women in Ireland who seek help from similar organizations. What these findings do offer, though, are early indications of both ACEs prevalence as well the types of ACEs that most define the experience of the women presenting to the domestic violence service; the next section identifies the central themes that emerged in terms of how this routine enquiry work with service users was performed in practice.

### Practice Themes

Given the routine enquiry results, the practitioner experiences of seeking to identify and respond to women's childhood trauma becomes key in how women might best be supported in their recovery from both substance use and domestic violence. Three key themes emerged from the dual inquiry group processes with practitioners; (1) discussing ACEs and responding to disclosure; (2) parental and personal substance misuse experiences in relation to earlier trauma; and (3) challenges for inter-agency work.

#### Discussing ACEs and Responding to Disclosure

The practitioners had initially queried whether, given their experience and expertise, an ACEs routine enquiry was needed to explore or discuss these issues of childhood trauma with women, but subsequently agreed that the ACEs questionnaire provided a useful framework:

Some of the women don't realize that their experience is good or bad, or that it has had such a profound impact on them. I often found the woman did not realize that their childhood experiences and current lifepaths are linked. (DV6)

For one practitioner, the ACEs routine enquiry helped refine what she described as her “traditional intuitive practice”:

As workers in the field, we know that childhood impacts women, but there's a big difference between having a suspicion and having a researched framework to put that in…and that deepens, certainly, my own practice. It's not anymore something I think, or intuitively feel when I support a woman…[that] she had a tough childhood, which we would so regularly do with our clients before…ACEs has put that framework on my traditional practice. (DV1)

Consideration was given to women who had low numbers of, or no ACEs, with the practitioners questioning whether there were other factors that may have impacted on health and relationships. They cited a number of cases where a single traumatic incident such as a random assault outside of the home or bullying by a teacher in school actually had a significant long-term impact on the women they worked with. There was also a general conclusion that the ACEs routine enquiry did not lend insight into the gendered cultural and societal expectations women may experience as children, though the ACEs routine enquiry did provide an opportunity to discuss this with women, at times providing a window of opportunity for assisting insight into life patterns:

Sometimes your work is so busy you don't get the opportunity to go back into the past or... because there's other issues that you're dealing with, but for one woman that I'm thinking about, well her mum, she came from an abusive relationship. Her dad was abusive to her and she (the woman) got animated and said, “Why did my mother stay there? She wasted 10 years of her life. Now look at me. I'm doing the exact same”. (DV6)

The practitioners also felt acknowledging a woman's past experiences and highlighting her emotional and practical strengths and resilience was really important, but also supporting her to see a different life path for herself, as one practitioner outlined:

One woman who ticked all the boxes, now she became really, really upset, but she already had identified that all those adverse child experiences had affected her life. She was really interested in resilience, and she got it that it didn't define her, which was really interesting - just looking at all of those questions, ticking all those boxes, she said it really reinforced that her childhood really affected her and how she has lived her life, how she has parented. But she can see that she can make changes. But it was like a clear picture for her. (DV8)

The practitioners highlighted a number of cases where women had older children (sometimes in care settings or dealing with the impact of their own ACEs), concluding that in such instances completing the ACEs routine enquiry should not reinforce guilt or shame. The workers agreed that guilt is an emotion that should be named and talked about, as it is so often a feature for women who have experienced domestic violence and substance use:

But I think guilt with domestic violence goes hand in hand. So again, even when you're supported, the guilt is going to be there regardless of staying for the children. Guilt is so key to domestic violence, never mind where there is substance use too. (DV2)

#### Agency Remit

The infant mental health practitioners were not engaged in a systematic implementation of ACEs routine enquiry, with some contemplating introducing it, and others who using it in a briefer format within client assessments. For the practitioners, the role and remit of their agency was a key determinative factor in whether they should consider integrating ACEs routine enquiry into their work. It was agreed that agencies needed to have client-centered practices; have the resources to support women effectively and have remit in regard to working with her and her children if she has them. As one practitioner highlighted, the purpose of the agency should influence the decision to consider implementing ACEs routine enquiry:

There has to be a consideration for the agency, about what's the purpose of this, so why would we do this as opposed to any other agency that those mothers might be in contact with. Well, she has a relationship with us and we think we could positively influence her parenting of her own kids or dealing with some issues in her life by doing that routine inquiry. (IMH3)

A recurring theme across the practitioners from all the agencies about implementing ACEs routine enquiry was the lack of certainty that resources would be there to respond to client issues that were raised. While referral pathways for counseling and other therapeutic interventions currently exist, it was felt these were limited, often with significant waiting lists or limits on the duration of the intervention. The infant mental health practitioners also debated whether the ACEs tool had value over other interventions or ways of considering trauma:

I think I would want to know, well, we have some understanding of why this would be more useful than just doing what we do–because I talk to women about these issues anyway. (IMH2)

#### Parental and Personal Substance Misuse Issues

A significant aspect for the practitioners was the degree and mechanisms by which the women articulated greater understanding and empowerment from the process of completing the ACEs routine enquiry and from the subsequent conversations, especially in relation to substance misuse. The practitioners agreed that the ACEs routine enquiry process often helped ameliorate the self-blame women felt–about their lives, their substance use and negative impacts on their own children–rather than increasing it, which had been an initial concern. One practitioner maintained that the understanding generated by ACEs routine enquiry opened a conversation about about self-blame for one woman as she was able tease out with how certain decisions and choices were made:

[She] took it on as empowering, as understanding, a self-understanding….It's the release of self-blame there that she feels: “There was a reason why I took this bad boyfriend I took that bad husband, I started drinking, I started using substances.” (DV5)

In a case where there was long term inter-related domestic violence and substance use, the practitioners agreed that not only was the process empowering for the woman, allowing her to advocate for her own children, but also allowed her to start to move on from her own experiences:

It put structure on her past, but it also gave her the strength to move forward. I think she argued it with social workers–that, “I am the way I am because of what happened to me and my childhood and now that's going to happen to my children if things don't change.” That was her argument. So, it was amazing to hear and watch that process taking place. Now she's a long way down the road in her process but it was great for her, and she actually said, which was massive, “I have to let go of the experiences that happened to me as a child because, how can I be a better mother if I don't let go of them?” (DV6)

This raises the question of how women may be supported to access further services and interventions, and the practitioners felt that the reflective conversations with women could increase motivation for positive change, In one case, according to the practitioner, there was an immediate link for the woman in terms of intergenerational patterns and substance use and she requested an appointment with the substance misuse service:

I would be very aware that her ACEs would be high. She had a seven score. I knew that it would be that at least, but her father was alcohol dependent, and she made that link, which was so quite amazing, between her father being a drinker, she herself would be alcohol dependent ….. Now I genuinely feel that link would not have happened because she didn't want her kids to see her like she saw her father, without her doing the ACEs routine enquiry first. (DV3)

For some of the practitioners, completing the ACEs routine enquiry had a profound effect on the woman they were working with:

She was like, “Oh, God. I'm ticking them all, nearly.” And then she was saying “Yes, I did have such a dysfunctional family and the pain I was in, and that all I ever wanted was to feel loved or to have someone to love me,” and how she ended up in domestic violence relationships trying to feel loved as well. And we spoke about the substance misuse being a reaction to all the pain she had. And it was really like, “I can see how my life went down that route because of what I've experienced as a child.” And this tool have really helped her to say, “Okay, I've had a dysfunctional family; it's been unbelievable. But by God, it's not going to happen to my children. That was then and this is now. I'm working so hard now.” I just found it extremely empowering for both of us. (DV5)

Given the practitioners were drawn from an infant mental health networking group, there was significant discussion around supporting mothers with new babies where the mother has substance use issues. There were concerns that women could be dealing with a lot already, even before the inclusion of ACEs routine enquiry. It was highlighted that women had often already provided some of the information on the ACEs questionnaire as part of their referral to some agencies, and if the referral is in regard to infant mental health and parenting issues, then the nature of the ACEs questionnaire might not be helpful:

Issues usually come up during the course of the conversations with parents, out of their story, and I wonder that if you push the stories, the questions too much, they would back off. (IMH 6)

However, the point was also made that the evidence base underpinning the ACEs questionnaire is helpful in advocating for children, where their mothers have completed a routine enquiry and her substance use had previously just been viewed as problematic:

It's very useful when you're fighting for services or you're seeking a case conference and you can say, well, yes we have the research now to back it up or this is the word to describe what this young child is going through. Or why this adult can't care for this child. So I find it very helpful to explain to people what it is because for a long time, people weren't giving it the attention it deserves. [Previously] she (the mother) was just the problem. (DV3)

#### Challenges for Inter-Agency Work

While the practitioners felt those in related agencies in the community would take women's experiences more seriously because of the evidence base evident in relation to ACEs, this was tempered by a concern that the ACEs routine enquiry could be implemented in agencies without important aspects of support, empowerment and follow-up. There were also concerns that a woman would be “reduced” to her ACEs score or would be requested to complete the routine enquiry within several agencies. It was felt the logistics needed to be worked out at inter-agency level so that the implementation would be effective. The practitioners also highlighted the importance of good training prior to implementation, and time and resources being allocated to practitioner skill development to ensure a positive experience for those being invited to complete ACEs routine enquiry:

I think that there is a risk that ACEs will broaden itself out. It might be better to keep ACEs within agencies that would give the woman the support and the acknowledgment of the trauma. If feels like its nearly at the point where people in the supermarket are doing ACEs enquiry. That's not good, in my opinion, there's risk. (DV4)

In particular the practitioners were concerned about the level of supports women might need after a disclosure and how an agency might enact a client-centered response:

If you provide or implement a framework which allows her to acknowledge some of her vulnerabilities, then you have a lovely piece of work and a responsibility to support her... but we need to think about how those vulnerabilities are perceived, and understood, and talked about to others. Because they're hers and she has a right to boundary them. (DV1)

The practitioners also voiced the concern that women may feel there were implications for acknowledging some childhood experiences and subsequent behaviors, depending on the remit of the agency:

And I wonder how effective and truthful the response would be if it is a social worker carrying it out, because it's just a different support session and people are going to be terrified of, if I tick this, what will this result in? (IMH5)

The practitioners had a wider, more philosophical discussion about the future of services delivery and how ACEs might fit into that. It was pointed out that it can be much harder for statutory agencies to innovate and introduce new practices. One practitioner drew the analogy of the statutory agencies being like a large tanker, given organizational infrastructures and numbers of staff, and that–with respect to making changes in approaches to working with and offering support services for families, children and trauma–it can take “nine miles for one of those tankers to turn or go back” (IMH1). Working with this analogy, the group discussed how smaller NGOs and community agencies can essentially act as smaller, more nimble boats providing more tailored family support. As the practitioner described:

I think if we were to go with this analogy, which is very powerful actually…There's people on the big ship [i.e., statutory agencies] who are looking to see what's happening and waiting for the turn to happen…[but] it's also empowering because… there's more mobile craft [i.e., smaller community agencies] that are starting to innovate and pick people up. (IMH1)

## Discussion

The routine enquiry results indicated ACEs to have a significant presence among women accessing the domestic violence service. Over one-half (58 per cent) of service users experienced two or more ACEs in their childhood and one-third (33 per cent) experienced four or more. Just 18 per cent of service users reported having experienced no ACEs in childhood. This high prevalence tracks with prior findings in the literature (11, 16) and it is important to note that these results are much higher than that of the general population samples accessing primary health care settings in previous studies (1). It is widely agreed that ACEs “scores” revealed in these screenings do not offer a causal link between ACEs in childhood and later life outcomes; rather, they are indications of both ACEs prevalence as well as the types of ACEs that most define the experience of the women presenting to a domestic violence service (18).

Given that the ACEs questions were primarily designed as a research tool, not a personal intervention tool (8), it falls to organizations and practitioners to consider its usefulness and then subsequently attend to developing an appropriate practice response. Practitioners from the range of agencies raised concerns related to “individualization” of women's ACEs scores and the provision of appropriate trauma informed responses to client disclosures. There were fears that women; “would become their ACE score;” that support services may not be adequately available to those who disclose traumatic experiences, or only for those who report a “high” ACEs score of more than 4; or that clients may be forced to re-tell their ACEs history to many different agencies. Child maltreatment in the form of verbal abuse was the most prevalent ACEs type, followed closely by physical abuse (of both the child and of other members in the household) and substance misuse (alcohol and/or drugs). While the ACEs “scores” revealed in these screenings do not offer a causal link between ACEs in childhood and later life outcomes, they do provide indications of both ACEs prevalence as well as the types of ACEs that most define the experience of the women presenting to the service.

An ever-present challenge–and one relevant for incorporating ACEs into trauma-informed care and community practice–is distinguishing between individual-level and group- or population-level application. Kelly-Irving and Delpierre (8) note that while the promotion of ACEs awareness in health, educational, and service settings is often both useful and commendable, placing a focus on an individual's ACEs score poses ethical questions and is a departure from the spirit of Felitti et al. (3). The risk is that the need for structural change, more health interventions and addressing of the determinants of health become obscured and individual level interventions privileged (8). Finkelhor [(49), p. 175] is careful to point out that “high ACEs scores…are not the same as trauma symptoms” and therefore cannot be taken as an indicative measure (on their own) for individual intervention. Therefore, both client-led responses and how agencies can work together to co-ordinate responses and interventions, while also protecting the privacy of service users, become key. In the absence of an emphasis on interpreting ACEs in a broader population-level context in the push for better integrated systems of care, prevention, and early intervention, individuals looking at their own ACEs scores may face fear over their future outcomes, stigma from others about their personal circumstances, and a burden of a now “individualized” problem. This was particularly pertinent when there was an intersection of domestic violence, substance use, and child welfare issues and concern of future requirements to disclose or not be able to control sharing of your ACEs information–most especially where child protection services may potentially be involved (50). For instance, could a high parental ACE score be utilized against a mother if there were child welfare concerns or if there were custody and access court proceedings? Kelly-Irving and Delpierre's [(8), p. 453] comment is pertinent, that ACEs should not “be used to incriminate parents, but rather reveal the conditions, particularly social conditions, in which parents and children live and how they cope.”

Practitioner skills in opening conversations about historical trauma in the lives of women with substance use histories has been found to be crucial (51). In this study, identifying experiences of parental substance use and/or their own substance use through the ACEs routine enquiry platform provided a basis for conversations and discussions about intergenerational patterns, positive change and resilience that may not have otherwise happened (51, 52). Given the intersection of domestic violence and substance use can be very challenging for a single agency to address (53), the ACEs routine enquiry was found to be a constructive and useful mechanism to make links with women about their past traumas and current life trajectories, including any misuse of substances. While the “simplicity” of the ACEs questionnaire was originally a concern for those implementing it, in practice, this became a strength. Once in use, the ACEs questionnaire was not viewed as an all-encompassing solution to address childhood legacies of trauma, but instead as a mechanism for opening a topic or aspect of a client's life patterns (21, 52).

The practitioners in this study were adamant the ACEs routine enquiry needed to be embedded into existing client-centered approaches (54), in order to avoid many of the challenges experienced by other agencies (33). The ACEs routine enquiry was utilized as a tool within relationships already based on empowerment and collaboration, which perhaps may differ from wider health or social service interventions (39). There were many examples of positive impacts from women completing the ACEs routine enquiry, with practitioners reporting the potential of ACEs to provide a simple and explainable framework for considering the impact of childhood experiences, a key challenge for addressing the needs of women dealing with intersectional issues (21). One important aspect was the potential for practitioners to work with women to address guilt and self- blame, particularly where she had children who had subsequently experienced ACEs. Igniting both desire and action to seek further supports to address the impact of childhood trauma for herself and for her children based on an understanding of past experiences is a complex process but one that can support recovery, and may shift practitioners beyond supporting women on day to day or single issues (53).

These are aspects of practice that may be difficult to capture in terms of quantifiable outcomes (55), and may require long-term trauma-informed interventions to address (56). Importantly, though, the impact on practitioners of support work needs to be attended to in the context of trauma-informed service delivery. The routine enquiry process revealed a significant prevalence of ACEs, both in terms of direct maltreatment and adverse home environments experienced as a child, among the service users of this organization. The practitioners here gave numerous examples of disclosures and subsequent conversations that had the potential to be both emotionally transformative for the client, but also emotionally impactful on the practitioner. This highlights an important question for those engaged in support work about both the boundaries and limitations of such work, and the impact on the practitioner of working with such issues (2). Ultimately, inter-agency responses may need to differentiate between being ACEs-aware vs. actively implementing ACEs routine enquiry within their own service and a recognition that deciding between these two paths must be based on the ability of the agency to both advocate for women and provide trauma-informed responses (21).

As with any practice-based innovation, change requires consideration of the evidence, development and implementation of an intervention, practitioner training and organizational support. The practitioners highlighted two aspects in particular that proved helpful for this work: the capacity of community agencies to access small, relevant funding streams for such work, and the ability of community agencies to be more flexible in relation to practice changes due to their size and remit. This suggests that NGOs and community organizations may be better placed to pilot or innovate practice changes where there are experiences of childhood adversity, and later domestic violence and substance use (57). This project was completed with a limited budget, but its successful execution benefited and relied upon on existing robust supervision and support structures within the host organization, and strong, pre-existing community and inter-agency relationships among all of the participating IMH practitioners. This network and infrastructure may not always be in place, which adds to funding considerations. Follow-on evaluations of impact and outcomes for practice change also require continuous funding streams that may potentially be separate from the original project funding but are of importance to understanding critical learnings for future endeavors (53).

## Conclusion

This research sought to identify the level of ACEs for women accessing a domestic violence service and explore both the enactment and responses by practitioners to ACEs routine enquiry. It also sought to consider the possibilities in regard to the use of ACEs routine enquiry with a range of practitioners who had an infant mental health remit. As such, this study was situated in a very specific setting. It took an action research approach to exploring the practice responses aspect of the project, collaboratively working with practitioners to build an understanding of the relevance, usefulness and responses to ACEs routine enquiry. The findings of this study offer considerations and implications for a number of groups: service users, practitioners, organizations (both individually and in an inter-agency context), funders, and future researchers.

This study has several limitations. The routine enquiry questionnaire has the potential to provide a level of insight into the prevalence and types of ACEs among domestic violence service users, however it is best suited to capturing exposure, but is not intended to make a causal link between ACEs experienced in childhood and subsequent life outcomes (8). The results in regard to practice responses are limited to the views of the practitioners, and do not include the views of the women who completed the ACEs routine enquiry, nor their perceptions of subsequent service responses. Action research is by its nature both generative and continuous, so often requires further cycles of research and inquiry particularly where there are complexity and emotional labor are key features of the research setting (58).

## Data Availability Statement

The raw data supporting the conclusions of this article will be made available by the authors, without undue reservation.

## Ethics Statement

The studies involving human participants were reviewed and approved by University College Dublin Human Research Ethics Committee. The patients/participants provided their written informed consent to participate in this study.

## Author Contributions

SM was PI on the study and oversaw, lead on research design, data collection and analysis, and writing up of all research outputs. MC analyzed the quantitative data and wrote up these results, as well as contributing to the literature review, discussion, and conclusion. MB contributed to the thematic analysis, writing up of the qualitative results, and discussion on this manuscript. All authors contributed to the article and approved the submitted version.

## Funding

This research was partially funded by TUSLA, the Child and Family Agency, Ireland. The funder provided a small grant to support the domestic violence agency to consider and explore the role of ACEs within practice development within the agency.

## Conflict of Interest

The authors declare that the research was conducted in the absence of any commercial or financial relationships that could be construed as a potential conflict of interest.

## Publisher's Note

All claims expressed in this article are solely those of the authors and do not necessarily represent those of their affiliated organizations, or those of the publisher, the editors and the reviewers. Any product that may be evaluated in this article, or claim that may be made by its manufacturer, is not guaranteed or endorsed by the publisher.

## Footnotes

^1^This includes living with a parent or family member who was depressed, suicidal, or experiencing mental health issues.
